# Informative Censoring—A Cause of Bias in Estimating COVID-19 Mortality Using Hospital Data

**DOI:** 10.3390/life13010210

**Published:** 2023-01-11

**Authors:** Hung-Mo Lin, Sean T. H. Liu, Matthew A. Levin, John Williamson, Nicole M. Bouvier, Judith A. Aberg, David Reich, Natalia Egorova

**Affiliations:** 1 Department of Population Health Science and Policy, Icahn School of Medicine at Mount Sinai, New York, NY 10029, USA; 2 Division of Infectious Diseases, Icahn School of Medicine at Mount Sinai, New York, NY 10029, USA; 3 Department of Anesthesiology, Perioperative and Pain Medicine, Department of Genetics and Genomic Sciences, Icahn School of Medicine at Mount Sinai, New York, NY 10029, USA; 4 Division of Parasitic Diseases and Malaria, Centers for Disease Control and Prevention, Atlanta, GA 30333, USA

**Keywords:** censoring, COVID-19, convalescent plasma

## Abstract

(1) Background: Several retrospective observational analyzed treatment outcomes for COVID-19; (2) Methods: Inverse probability of censoring weighting (IPCW) was applied to correct for bias due to informative censoring in database of hospitalized patients who did and did not receive convalescent plasma; (3) Results: When compared with an IPCW analysis, overall mortality was overestimated using an unadjusted Kaplan–Meier curve, and hazard ratios for the older age group compared to the youngest were underestimated using the Cox proportional hazard models and 30-day mortality; (4) Conclusions: An IPCW analysis provided stabilizing weights by hospital admission.

## 1. Introduction

From March through June of 2020, New York City experienced a surge of coronavirus disease (COVID-19). The novelty of this illness limited treatment options and resulted in significant morbidity and death. In-hospital mortality has become a frequently described endpoint in COVID-19 clinical studies. Calculating this outcome requires multiple patient-level and hospital-level variables that have been difficult to trace during this pandemic. Many previously healthy people were hospitalized with COVID-19 and rapidly fell ill with slowly declining trajectories. Mortality often occurred after a prolonged index admission or upon subsequent readmissions. Patient transfers were common, since many patients became ventilator-dependent and intensive care units reached their capacities. Moreover, recovering patients were transferred to temporary intermediate care sites, such as the one at the Jacob Javits Convention Center in New York City, to make room in hospitals for incoming COVID-19 patients. Moribund patients were often transferred to out-of-system hospice and long-term care facilities in order to free up space on the wards. Electronic medical records between transferring facilities were frequently incompatible, and post-discharge expiration was not always reported back to the index facility. Therefore, the ultimate outcomes of many discharged patients cannot be determined from the primary hospital’s medical records. Wolkewitz et al. and Yi et al. [1,2] discussed different types of bias that may occur when analyzing in-hospital COVID-19 data.

Time-to-event analysis, also called survival analysis, models patients’ survival probability during their hospitalization. This analysis relies on three possible endpoints at a given time point, including “inpatient death”, “still hospitalized”, and “discharged alive”. If one of these events does not occur during the pre-specified follow-up time or a patient is lost to follow-up, the patient is then “censored.” Censoring patients with COVID-19 occurred when patient data was no longer available post discharge from the primary facility. The reason for censoring is that one cannot infer outcomes as the data are unavailable. The major assumption of this standard survival methodology is that the censoring is non-informative, meaning that patients’ withdrawals from a study are assumed to be independent of their characteristics and failure times. If the study cohort is a mixture of subpopulations determined by some implicit factors, then these factors might influence the timing and the probability of whether a patient will be lost to follow-up. For example, age can influence censoring. If younger COVID-19 patients tended to be discharged much earlier, then the remaining cohort would be older and may have an inherently increased risk of death. This violates the assumption that the discharged patients and the patients who remain hospitalized all follow the same survival distribution. This non-informative censoring assumption (e.g., censoring is not associated with cohort attributes) may lead to gross overestimates or underestimates of inpatient mortality.

Depending on the analytic approach, informative censoring may lead to biased survival estimates [3,4]. An increasing awareness of this issue appears in a variety of medical literature involving patients with COVID-19 [1,2], surgical site infections [5], kidney transplants [6], and cancer [7]. Simulation studies demonstrate that the magnitude of the bias depends on the hazard ratio of the treatment effect and the proportion of patients who are informatively censored [8]. If the patients who are informatively censored are at higher risk of an event than those who remain in the study, then the survival probability would be overestimated. Relative bias increases proportionally with an increasing number of informatively censored patients. Thus, a larger sample size will not remove the bias that is incurred with informative censoring.

There is no standard approach to control for informative censoring in survival analysis methodology. One proposed approach accounts for factors that affect event times and censoring times [9]. For example, antivirals [10] and steroids [11] have been suggested to improve hospitalized patient survival. Conversely, metabolic complications of COVID-19, such as established or newly diagnosed diabetes, is associated with increased mortality [12]. Including these COVID-19 directed treatment options and concomitant treatments for associated COVID-19-associated complications as time-varying covariates can help modify the confounding effect. A second recommendation requires a sensitivity analysis to gauge the effect of informative censoring on the outcomes. A competing risk analysis can also be applied to independently model different causes of clinical outcomes [13,14]. However, it is important to understand the distinction between a competing risk and a censored observation. A competing risk is an event whose occurrence precludes the occurrence of the event of interest. For example, performing a tracheostomy on an intubated patient may increase their survival. The risk of receiving a tracheostomy (as an outcome) is therefore a competing risk for mortality (as another outcome). Censoring, on the other hand, refers to an inability to determine the time at which an event occurs. If a patient was discharged and subsequently received a tracheostomy at another facility, that information would be unknown. As noted by Wolkewitz et al. [1], cumulative incidence functions should be used instead of Kaplan–Meier survival curves when analyzing in-hospital data, accounting for discharge as a competing event. Here, the inverse probability of censoring weight (IPCW) approach is offered [15,16,17].

IPCW accounts for the mechanism of censoring when performing a survival analysis. IPCW adjusts parameter estimates with the conditional probabilities of being uncensored for each patient throughout the follow-up period. IPCW estimators correct for informative censoring by allocating additional weight to subjects who are not censored. The scope of this paper will not describe the IPCW method in detail, but rather introduces this method as an advantageous solution to the informative censoring dilemma in survival analysis for any group of patients with personal or systemic characteristics that make them difficult to follow—here, specifically COVID-19 patients.

## 2. Materials and Methods

To illustrate how IPCW works, suppose there are 100 hospitalized COVID-19 patients on admission (day 0) and 80 of them were discharged alive before day 30. In a typical survival analysis, the risk set of patients who could potentially die on day 30 only includes the 20 subjects who remained in the hospital. If all patients were kept in the hospital even after their recovery, there would have been 80 extra subjects at risk of death. By weighting the contribution of subjects who are not discharged by the inverse of the proportion of the remaining patients (1/(20/100) = 5), the risk set changed from 20 to 100. Hence, the censored patients were analyzed as if they were included in the computations to estimate the survival rate. Since the conditional probabilities of being censored increase with time, the weights similarly increase with time.

Uncensoring probability (the chance of remaining at risk of death in the study, opposite of discharged alive) is denoted as pi(t) for subject i at time t, given that the subject is still in the study at time t−1.

The conditional probability that the patient remains uncensored up to time t (i.e., died on time t) is

pi(1) × pi(2) × … × pi(t)(1)

The weight at time t for subject i is

wi(t) = 1/[pi(1) × pi(2) × … × pi(t)](2)

This weight can become very large, which could lead to instability in parameter estimation. Alternatively, a stabilized weight is often used by incorporating a term to the numerator for each 1/pi(t).

The final weight becomes

wi(t) = [si(1) × si(2) × … × si(t)]/[pi(1) × pi(2) × … × pi(t)](3)

Informally, the weight is a ratio of a patient’s probability of remaining uncensored up to day t, calculated as if there had been no time-dependent determinants of censoring, divided by the subject’s conditional probability of remaining uncensored up to day t.

**Figure 1:**
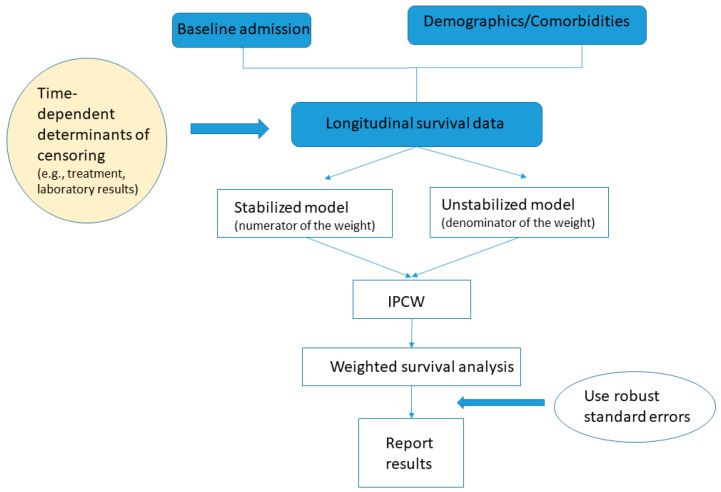
Schematic diagram for the inverse probability of censoring weighting analysis.

The practical question is how to obtain wi(t). The first step is to build two logistic regression models to estimate the probability of remaining uncensored on a given time t, one of which includes only baseline characteristics and is used to construct si(t) in the numerator of the weight (also known as the stabilized model) and the other also includes time-varying covariates and is used to construct pi(t) in the denominator (also known as the un-stabilized model). The fundamental difference between the two models is the incorporation of the time-dependent covariates in estimating pi(t). Without the time-varying covariates, the si(t)/pi(t) = 1 for all patients, which would imply that the probability of uncensoring is independent from survival time. Therefore, by adding the time-dependent covariates, the informative censoring as a function of survival time is captured. Specifically, before building the logistic regression models, a new observation that includes all of the covariates under consideration and survival outcome should be entered into the dataset either for each follow-up day or whenever there is a change in the covariate profile during follow-up (e.g., adding a new antibiotic drug to the existing treatment regimen). In addition, the cumulative follow-up duration as well as the period indicator (calendar time during the pandemic) should also be included. The resulting longitudinal dataset reflect the change in the patient’s treatment history and health condition, which can then be used to predict the temporal probability of remaining in the hospital versus being discharged alive. Once the models are built, the second step is to estimate the weight wi(t) as described above. The last step would be to use standard survival software with the weight option to perform survival analysis that allows repeated events. It is important to be aware that the standard errors of the parameter estimates from the typical software output tend to be underestimated due to the fact that the weights are estimated with uncertainty. Robust (empirical) standard errors should be used instead. A schematic diagram of the method is shown in Figure 1.

To illustrate the method, retrospective data for patients within the Mount Sinai Health System (MSHS) in New York City who were treated with SARS-CoV-2 convalescent plasma is analyzed. This study was approved by Institutional Review Board of Icahn School of Medicine at Mount Sinai. Time 0 was defined as the time of the first admission when the COVID-19 test was confirmed positive (index admission). The measured outcome was mortality.

For the plasma example, the final outcome of interest is the survival after the index admission. To construct the stabilizing weights, two logistic regression models were built to predict the probability of not being discharged alive for a given hospitalization, without and with time-varying covariates, respectively. Specifically, the covariates included the admission hospital site, age, sex, race and ethnicity, week of calendar time, and duration of follow-up in the stabilized logistic regression model for si(t). In particular, a restricted cubic spline curve was fit for week and length of cumulative follow-up time since the index admission. The cubic spline curve allows for a non-linear relationship between a time-related variable and the logit of the probability of remaining at risk of death. The un-stabilized logistic regression model for pi(t) considered the above covariates and the following time-dependent covariates, which included whether the patient received any of the following interventions during the visit: plasma treatment status, intubation and tracheostomy status, use of investigational and/or off-label drugs (e.g., investigational antiviral, hydroxychloroquine, and azithromycin), and use of therapeutic anticoagulants, broad-spectrum antibiotics, antiplatelet agents, and steroids. For the purpose of illustration, here, the number of observations used in the logistic regression models for each patient was the number of hospital admissions (see Discussion). Hence, the covariates differed by hospital admissions, but remained the same within the same hospitalization. In the last step of IPCW survival analysis, weight is incorporated into the standard survival software model. As some patients were readmitted multiple times after their initial discharge, a counting process approach was used to analyze the multiple admission data and reported empirical standard errors for parameter estimates (see Supplementary Materials for SAS code).

## 3. Results

Between 24 March 2020 and 14 July 2020, 441 patients hospitalized at five hospitals within the MSHS received convalescent plasma. At the time of analysis on 14 July 2020, 133/441 (30.0%) of patients had died and 14 patients were still in the hospital. The majority (n = 378) of patients had a single admission; however, 63 patients were readmitted after their initial admission, for a total of 524 unique admissions (Table 1). The median follow-up time was 13 (IQR 18; max 97) days for patients who were discharged alive, 18 (IQR 19; max 90) days for patients who died, and 94 (IQR 23; max 112) days for patients remaining in the hospital. Of the 133 deaths, six occurred in the subsequent readmissions after the plasma therapy (1.4%). Table 1 shows the descriptive statistics for the cohort, stratified by the number of admissions.

### 3.1. Outcomes with Reduced Bias

#### 3.1.1. Overall Mortality

When patients who were discharged alive were treated as censored observations at the time of discharge, and patients who remained hospitalized were treated as censored observations on 14 July, the unadjusted Kaplan–Meier curve suggests that half of the patients would have died before week 6, and the mortality rate would be >60% by week 9 (unadjusted scenario). However, if those who were discharged were all still alive as of 14 July, the 90-day mortality post COVID-19 diagnosis would be 133/441 = 30% (best case scenario). In reality, among those who left the hospital, the discharge disposition included a hospice or long-term care facility. Therefore, the true 90-day mortality should be close to, but greater than, 30%.

**Figure 2:**
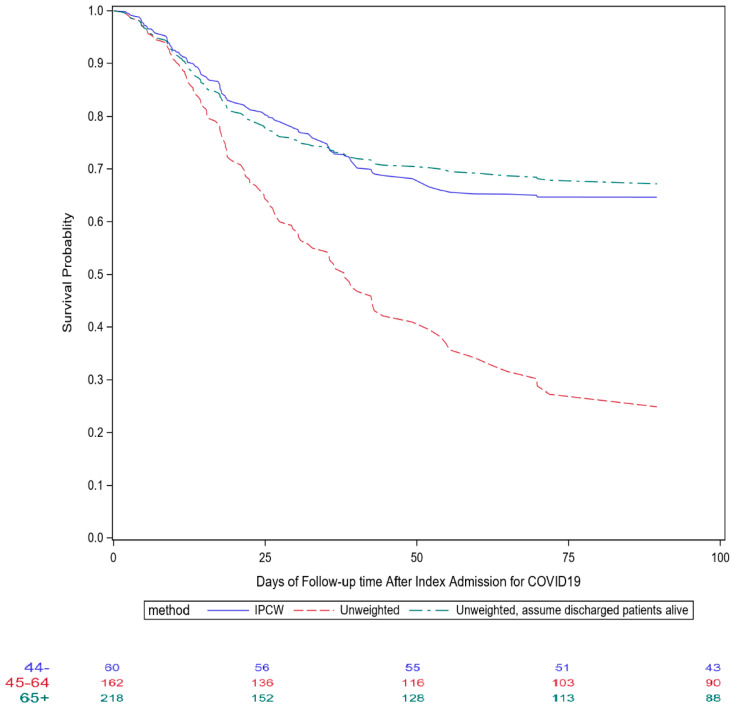
Comparisons of different survival curves after index admission for COVID-19 for patients who received convalescent plasma therapy in Mount Sinai Health System.

Figure 2 depicts the survival curves estimated using three different approaches: the unweighted approach for recurrent admission events, the same unweighted approach assuming all discharged patients were still alive on 14 July (best-scenario approach for sensitivity analysis), and the IPCW approach.

#### 3.1.2. Age Group Comparisons

For an illustration of how informative censoring affects the group comparison, Table 2 shows the mortality hazard ratios for the age groups 45–65 and >65 years old, relative to the younger than 45 years old age group, using the Cox proportional hazard models under the three scenarios as shown in Figure 2. Additionally, the competing risk analysis that treated hospital discharge as a competing outcome was also performed. All four models suggest that the risk of mortality increases as age increases. The largest difference was seen for the 65 years and older vs. younger than 45 years age group comparison between the regular Cox model (HR = 3.19) and the other two models (HR = 5.44, 5.87 and 5.94, for the IPCW model, competing risk model, and the best scenario model, respectively). Younger patients were more likely to be discharged early, resulting in an increasing older and sicker cohort as follow-up time lengthened. Therefore, the regular Cox model under-estimated the true hazard ratio for the 65+ group.

#### 3.1.3. 30-Day Mortality

Our institution ascertains the death status for all hospitalized patients using the Social Security Administration Limited Access Death Master File (LA-DMF, updated monthly) and LexisNexis database (updated weekly). As of 15 August, only one additional death was found after final hospital discharge, which led to an overall mortality rate of 30.2%, almost identical to the rate estimated by the “best scenario” approach described above. Except for those patients who died, all patients had had at least 30 days of follow up after index admission, with a 30-day mortality of 24.0% (95% CI: 20.0–28.3%). In comparison, the estimated 30-day mortality was 43.1% (34.9–48.5%) for the regular unweighted method, 24.6% (20.3–28.6%) for the unweighted best scenario approach, and 22.8% (IPCW, 95% CI: 14.6–30.2%) for the IPCW approach. Furthermore, the relative risk of 30-day mortality based on proportions of patients who died was 2.43 (0.95–6.25) and 5.36 (2.01–14.29) for those aged 45–64 and 65+, respectively, when compared to the under 45 age group.

#### 3.1.4. Influence of Time-Dependent Covariates

To investigate and compare the influence of the time-dependent covariates on the estimation of age effects in Table 2, we performed a series sensitivity analysis. The first analysis was to include the time-dependent treatment covariates only in the denominator of the weight and set the numerator of the weight to be 1 (Table 2). The impact of older age on mortality was significantly greater. Because the older patients tended to receive more and longer treatment interventions, the age effects were confounded by the time-dependent treatment covariates in the unadjusted regular Cox model.

The second analysis was to omit one time-dependent variable at a time in estimating the marginal probability of remaining uncensored on a given day (Table 3). The largest differences, compared to the IPTW final results in Table 2, were seen for the intubation status and convalescent plasma status. The durations of mechanical intubation and convalescent plasma treatments were significantly correlated with age, as well as whether a patient remained in the hospital. For example, patients who had an extended hospital stay tended to be intubated and older. Therefore, the failure to account for this led to a similar finding to that of the unadjusted regular Cox model.

## 4. Discussion

An awareness of the non-informative censoring problem should prompt caution when analyzing COVID-19 data, particularly when using hospital data. Unfortunately, discharged participants are often lost to follow up unless state- or national-level death data are available. The LA-DMF, which is available for download and incorporation into local hospital registry data, is known to be incomplete because of restrictions imposed on the sharing of state-level data [18]. State-level mortality queries require individual data use agreements with each state of interest (EX SPARCS, https://www.health.ny.gov/statistics/sparcs/access/, accessed approximately 15 August 2020 [19]. The Centers for Disease Control National Death Index [20] has significant restrictions on access and use that make it unsuitable for incorporation into time-sensitive analyses (https://www.cdc.gov/nchs/ndi/index.htm, accessed approximately 15 August 2020 Therefore, local hospital data are often used to estimate overall mortality for hospitalized patients.

When treating discharged-alive patients as censored observations at the time of discharge, large numbers of early censored values decrease the number of subjects at risk of death at later times, reducing the effective sample sizes. Heavy censoring may also be indicative of a pattern in the censoring, including younger and healthier patients being discharged earlier, leaving only older and more sick patients (for example, those with a prolonged time on a ventilator) who represent a very high-risk subset. This accelerates the drop of the Kaplan–Meier survival curve, particularly towards the later part of the follow-up period.

A popular alternative for analyzing mortality data commonly seen in the medical literature is to model death as a binary outcome using logistic regression. It is used in the context of a clear and well-defined study period, such as 30-day mortality. The follow-up duration is often short so that the final outcome can be ascertained for every subject by the study end without any censored observations. In addition, the mortality rate is expected to be low. A log-binomial regression model for relative risk might be used when the mortality rate is greater than 10%. For hospitalized patients with COVID-19, the overall in-hospital mortality was estimated to be more than 20% in our institution as of 14 July (internal data). Neither approach is able to depict how the risk of mortality changes throughout the follow-up period. The timing of death is important for patients with COVID-19 in order to determine treatment options, as most severe adverse events happen later during admission rather than earlier.

This study has some important limitations. The convalescent plasma example is used to demonstrate the potential and neglected bias in estimating survival outcomes due to informative censoring. In the analysis, only those patient and clinical factors with the best data quality were considered for illustration. There are other clinical and comorbidity variables that also affect the survival outcomes for patients with COVID-19. The goal of the current analysis is not to establish whether convalescent plasma is effective, nor to identify the factors associated with plasma survival. In addition, the data were only representative of one health system in the New York City metropolitan area collected during the peak of the local epidemic.

As demonstrated in Figure 2, the unadjusted approach substantially underestimated the survival probability, whereas the IPCW approach yielded a similar survival curve as the curve that assumed all discharged patients were still alive, with slightly lower 90-day mortality towards the end of the follow-up. In the IPCW analysis, we constructed the stabilizing weights by hospital admission. Therefore, the weights would vary depending on the duration of hospitalization, number of hospitalizations, treatment regimen, and patients’ medical and baseline characteristics. Because most aforementioned therapeutic interventions were given shortly after admission, this assumption might be reasonable, but overly simplified. An improved analytic approach would be to enter one observation for each follow-up day to take into account the daily variation in patient’s health status, reflected by laboratory test results, vital signs, and invasive oxygen treatment.

## 5. Conclusions

During the first wave of the COVID-19 pandemic, many studies attempted to estimate the true mortality rate from COVID-19. This raises the concern of a neglected cause of bias in estimating COVID-19 mortality using hospital data, which arises from treating the discharged patients as typical censored observations in survival analysis. Some recommended remedies are briefly discussed, with a focus on the IPCW method because it is a less familiar approach in the medical literature. As the plasma example showed, the Kaplan–Meier curve, especially towards the later follow-up period, vastly overestimated the mortality rate. In our plasma example, as almost all patients now have been discharged, the picture of the true in-hospital mortality is more attainable. Therefore, the biased estimate of the survival rate using the Kaplan–Meier method becomes more obvious. The clinical implications of this method allow for an improved accuracy of true mortality rates and treatment effects derived from observational data. Hence, clinicians and researchers should be aware of the impact of informative censoring when reviewing the earlier COVID-19 publications. In planning future analyses using COVID-19 in-patient data, it is important to incorporate not only baseline characteristics data, but also to document time-dependent vital sign and laboratory data, as well as the timing of different treatment regimens. These data are crucial for model adjustment and for understanding the censoring mechanism. Finally, all analyses should be accompanied by a thorough sensitivity analysis to understand the degree of bias, if any, present in the selected models.

## Figures and Tables

**Table 1:** Baseline and in-hospital characteristics for the convalescent plasma recipients.

Admission Visit Number before 7/15/2020								
Covariate	Statistics	Level	1 N = 378	2 N = 47	3 N = 12	4 N = 4	Parametric *p*-Value *	Non-Parametric *p*-Value **
Discharge Disposition	N (Row %)	Discharged Alive	242 (82.3)	40 (13.6)	8 (2.7)	4 (1.4)	<0.001	<0.001
	N (Row %)	Expired	126 (94.7)	6 (4.5)	1 (0.8)	0 (0)		
	N (Row %)	In Hospital	10 (71.4)	1 (7.1)	3 (21.4)	0 (0)		
Hospital Site	N (Row %)	A	245 (84.2)	33 (11.3)	9 (3.1)	4 (1.4)	0.960	1.000
	N (Row %)	B	37 (84.1)	5 (11.4)	2 (4.6)	0 (0)		
	N (Row %)	C	37 (90.2)	4 (9.8)	0 (0)	0 (0)		
	N (Row %)	D	31 (91.2)	3 (8.8)	0 (0)	0 (0)		
	N (Row %)	E	28 (90.3)	2 (6.5)	1 (3.2)	0 (0)		
Sex	N (Row %)	F	154 (85.1)	21 (11.6)	3 (1.7)	3 (1.7)	0.327	0.360
	N (Row %)	M	224 (86.2)	26 (10)	9 (3.5)	1 (0.4)		
Race/Ethnicity	N (Row %)	Asian	30 (81.1)	6 (16.2)	1 (2.7)	0 (0)	0.209	0.193
	N (Row %)	Black	73 (86.9)	11 (13.1)	0 (0)	0 (0)		
	N (Row %)	Hispanic	96 (81.4)	13 (11.2)	6 (5.1)	3 (2.5)		
	N (Row %)	Other	105 (92.1)	7 (6.1)	2 (1.8)	0 (0)		
	N (Row %)	White	74 (84.1)	10 (11.4)	3 (3.4)	1 (1.1)		
Therapeutic Anticoagulant	N (Row %)	No	103 (74.6)	27 (19.6)	6 (4.4)	2 (1.5)	<0.001	<0.001
	N (Row %)	Yes	275 (90.8)	20 (6.6)	6 (2.00)	2 (0.7)		
Broad Spectrum Antibiotics	N (Row %)	No	123 (75)	33 (20.1)	6 (3.66)	2 (1.2)	<0.001	<0.001
	N (Row %)	Yes	255 (92.1)	14 (5.1)	6 (2.17)	2 (0.7)		
Anti-Platelets	N (Row %)	No	273 (84.5)	38 (11.8)	9 (2.8)	3 (0.9)	0.656	0.672
	N (Row %)	Yes	105 (89.0)	9 (7.6)	3 (2.5)	1 (0.9)		
Steroids	N (Row %)	No	162 (78.3)	32 (15.5)	11 (5.3)	2 (1.0)	<0.001	<0.001
	N (Row %)	Yes	216 (92.3)	15 (6.4)	1 (0.4)	2 (0.9)		
Tocilizumab	N (Row %)	No	346 (85.0)	45 (11.1)	12 (3.0)	4 (1.0)	0.488	0.668
	N (Row %)	Yes	32 (94.1)	2 (5.9)	0 (0)	0 (0)		
Investigational Antiviral	N (Row %)	No	323 (84.1)	45 (11.7)	12 (3.1)	4 (1.0)	0.094	0.106
	N (Row %)	Yes	55 (96.5)	2 (3.5)	0 (0)	0 (0)		
Hydroxy- chloroquine	N (Row %)	No	182 (76.5)	41 (17.2)	11 (4.6)	4 (1.7)	<0.001	<0.001
	N (Row %)	Yes	196 (96.6)	6 (3.0)	1 (0.5)	0 (0)		
Azithromycin	N (Row %)	No	193 (78.5)	39 (15.9)	10 (4.1)	4 (1.6)	<0.001	<0.001
	N (Row %)	Yes	185 (94.9)	8 (4.1)	2 (1.0)	0 (0)		
Mechanical Ventilation	N (Row %)	No	296 (82.9)	46 (12.9)	11 (3.1)	4 (1.1)	0.006	0.002
	N (Row %)	Yes	82 (97.6)	1 (1.2)	1 (1.2)	0 (0)		
Tracheostomy	N (Row %)	No	357 (85)	47 (11.2)	12 (2.9)	4 (1.0)	0.299	0.408
	N (Row %)	Yes	21 (100)	0 (0)	0 (0)	0 (0)		
Plasma Therapy	N (Row %)	No	0 (0)	37 (75.5)	9 (18.4)	3 (6.1)	<0.001	<0.001
	N (Row %)	Yes	378 (96.4)	10 (2.6)	3 (0.8)	1 (0.3)		
Age	Mean		63.0	62.9	63.8	52.8	0.595	0.841
	Median		64	65	66.5	59		
	Min		19	23	44	20		
	Max		96	96	89	73		
	Std Dev		14.8	16.2	13.0	23.0		
Days to Plasma Therapy from Index Admission	Mean		4.8	5.2	13.6	27.3	<0.001	0.080
	Median		2.4	2.5	4.6	13.1		
	Min		0.1	0.9	0.4	1.9		
	Max		48.3	34.6	67	81.2		
	Std Dev		7.5	6.5	20.6	37.3		

* The parametric *p*-value is calculated by ANOVA for numerical covariates and chi-square test for categorical covariates. ** The non-parametric *p*-value is calculated by the Kruskal–Wallis test for numerical covariates and Fisher’s exact test for categorical covariates.

**Table 2:** Hazard ratios for age group among convalescent plasma recipients.

Model	Age Group	Hazard Ratio	95% CI *		*p*-Value *
Regular Cox Model	45–64 vs.<45	1.94	0.79	4.74	0.147
	65+ vs. <45	3.19	1.34	7.60	0.009
IPCW Cox Model	45–64 vs. <45	1.81	0.50	6.51	0.362
	65+ vs. <45	5.44	1.73	17.06	0.004
IPCW Cox Model with Only Time-Dependent Treatment Covariates	45–64 vs. <45	2.59	0.61	11.09	0.473
	65+ vs. <45	7.54	2.06	27.57	0.001
Competing Risk Model	45–64 vs. <45	3.35	1.34	8.40	0.010
	65+ vs. <45	5.87	2.40	14.33	0.001
Regular Cox Model, Assuming Discharged Patients Were Still Alive on 14 July 2000	45–64 vs. <45	3.42	1.36	8.59	0.009
	65+ vs. <45	5.94	2.43	14.53	<0.0001

* Based on empirical standard errors.

**Table 3:** Sensitivity analysis of Table 2 analysis for omitting the time-dependent covariate in building the marginal probability of remaining uncensored.

Time-Dependent Variable Omitted	Age Group	Hazard Ratio	95% CI *		*p*-Value *
Therapeutic Anticoagulants	45–64 vs.<45	1.69	0.44	6.53	0.450
	65+ vs. <45	5.90	1.83	19.07	0.003
Broad-Spectrum Antibiotics	45–64 vs. <45	1.67	0.43	6.497	0.463
	65+ vs. <45	5.64	1.69	18.76	0.005
Antiplatelet Agents	45–64 vs. <45	1.76	0.48	6.40	0.390
	65+ vs. <45	5.40	1.73	16.83	0.004
Steroids	45–64 vs. <45	1.79	0.48	6.70	0.386
	65+ vs. <45	5.77	1.82	18.36	0.003
Investigational Antiviral	45–64 vs.<45	1.87	0.54	6.53	0.326
	65+ vs. <45	4.84	1.51	15.50	0.008
Hydroxychloroquine	45–64 vs.<45	2.01	0.56	7.28	0.286
	65+ vs. <45	5.97	1.88	19.01	0.003
Azithromycin	45–64 vs.<45	1.78	0.49	6.42	0.378
	65+ vs. <45	5.37	1.72	16.77	0.004
Plasma Treatment Status	45–64 vs.<45	3.42	1.02	11.43	0.046
	65+ vs. <45	7.33	2.22	24.18	0.001
Intubation Status	45–64 vs.<45	2.23	0.77	6.47	0.138
	65+ vs. <45	3.41	1.37	8.48	0.008
Tracheostomy Status	45–64 vs.<45	1.76	0.48	6.41	0.392
	65+ vs. <45	5.31	1.68	16.75	0.004

* Based on empirical standard errors.

## Data Availability

Data will be shared in accordance with ISMMS policy on access to and use and disclosure of Mount Sinai data. This process can be initiated upon request to the corresponding author.

## Supplementary Materials

The following supporting information can be downloaded at: https://www.mdpi.com/article/10.3390/life13010210/s1.
