# Supplementary material for: Informative Censoring—A Cause of Bias in Estimating COVID-19 Mortality Using Hospital Data
Source: Life (Basel). 2023 Jan 11;13(1):210. doi: 10.3390/life13010210 (PMC9865049; doi:10.3390/life13010210)
Supplement: Supplementary file 1 [file life-13-00210-s001.zip › life-2064840-supplementary.pdf]

e-Supplement:

For the purpose of illustration, here the number of observations used in the logistic regression models for each patient was the number of hospital admissions. Hence, the covariates differed by hospital admissions, but remained the same within the same hospitalization. Because most aforementioned therapeutic interventions were given shortly after admission, this assumption might be reasonable, but overly simplified. Alternatively, an improved analytic approach was to enter one observation for every follow-up day to take into account the daily variation in patient's health status, reflected by laboratory test results, vital signs and invasive oxygen treatment.

```
/***********************************************************************************

** Step 1: Construct models to estimate weight

Stabilized logistic regression model for si(t) includes study site (site), age, gender, race and ethnicity (race_eth), week of calendar time for the index admission (week), and duration of follow-up (cum_fu_days). In particular, we fit a restricted cubic spline curve for week and cumulative follow-up time since index admission to allow non-linear relationship between time and the logit of the probability of remaining uncensored.

Unstabilized logistic regression model for pi(t) also considers the above covariates and time-dependent covariates, including plasma status and time to plasma therapy since index admission (plasma_now and days_to_plasma), intubation and tracheostomy status and time to start intubation or tracheostomy since index admission(intub_now, trach, days_to_intub, and days_to_trach), use of investigational and/or off-label drugs (tocilizumab, investigational antiviral, hydroxychloroquine, azithromycin), and use of therapeutic anticoagulant (ac), broad spectrum antibiotics (abx), antiplatelet (antiplt), and steroids.

***********************************************************************************/

** Stabilized model:  baseline covariates only;

proc logistic data=long_demo_dx_ descending;
class gender race_eth;
effect cum_fu_days_SP = spline(cum_fu_days / basis=tpf(noint) naturalcubic
knotmethod=percentiles(3) details);
effect   week_SP = spline(week / basis=tpf(noint) naturalcubic
knotmethod=percentiles(3) details);
model not_discharged = Site  age gender  race_eth week_SP cum_fu_days_SP;
output out=cp p = censor_p;


** Unstabilized model: with time dependent covariates;

proc logistic data=long_demo_dx_ descending; class gender race_eth bmi_cat site renlfail_c;
effect cum_fu_days_SP = spline(cum_fu_days / basis=tpf(noint) naturalcubic
knotmethod=percentiles(3) details);
effect   week_SP = spline(week / basis=tpf(noint) naturalcubic
```

```
knotmethod=percentiles(3) details);
effect LOS_days / basis=tpf(noint) naturalcubic knotmethod=percentiles(3) details);
model not_discharged =            above list  +
                                  Plasma_now days_to_plasma
intub_now  days_to_intub
trach  days_to_trach
ac  steriods abx antiplt ac abx antiplt steriods
                          tocilizumab antiviral hydroxychloroquine azithromycin
week_SP  cum_fu_days_SP LOS_days_SP ;
output out = ctp p = censor_tp;


/*************************************************************************************

** Step 2: Calculate the weight
*************************************************************************************/

data cp;
merge ctp cp; by mrn;
retain cwt;
r = (censor_p)/(censor_tp);
if first.mrn then cwt = r; else  cwt = cwt*r;


/*************************************************************************************

** Step 3: Estimate survival probability
```

The example used here is for Table 2 analysis. We used the counting process approach, such that the weight cwt within the interval (start_day, end_day) is the same.

The covs(aggregate) option with the id statement will generate the empirical standard error.

```
*************************************************************************************/

data Inrisks;
  length Age $20;
  input age_grp  Age $;
  datalines;
1  45-
2  45-64
3  65+
;

proc phreg data = cp covs(aggregate)  plots(overlay)=survival;
id mrn;
model (start_day, end_day)*death(0)= Age_grp /ties=efron rl;
weight cwt;
baseline covariates=Inrisks out=Pred_IPCW survival=_all_/rowid=Age;
run;
```
